# Relationship between spirituality and humanized care in nursing professionals at a Chilean hospital

**DOI:** 10.1590/0034-7167-2024-0598

**Published:** 2025-12-15

**Authors:** Daniela Jacqueline Espinoza-Padilla, Margarita del Carmen Poblete-Troncoso, Verónica Teresa Guerra-Guerrero, Cláudia Zamberlan, Dirce Stein Backes

**Affiliations:** I Hospital Dr. Franco Ravera Zunino. Rancagua, Chile; II Universidad Católica del Maule, Centro de Investigación del Cuidado. Talca, Chile; III Universidade Franciscana. Santa Maria, Rio Grande do Sul, Brazil

**Keywords:** Spirituality, Humanization of Care, Nursing, Nursing Care, Public Hospitals., Espiritualidad, Humanización de la Atención, Enfermería, Atención de Enfermería, Hospitales Públicos.

## Abstract

**Objectives:**

to analyze the relationship between spirituality and humanized care among nursing professionals in a Chilean hospital.

**Methods:**

observational, analytical, and cross-sectional study with a quantitative approach carried out with 119 nursing professionals from a hospital. Data were collected between April and July 2024, through the application of the following questionnaires: bio-sociodemographic, Spirituality SQ, and Nyberg’s Caring Assessment. The SPSS version 25.0 program was used for data analysis.

**Results:**

average levels of spirituality and humanized care were found, which reflects that nurses have interpersonal skills to provide holistic care; in addition, there is a significant statistical correlation (p≤0.05) between the constructs.

**Conclusions:**

spirituality determines humanized care through an intrinsic ethical value, as indicated by Jean Watson’s Theory of Human Caring. Therefore, hospitals should consider these elements in the training and practice of nursing professionals to provide a more holistic care.

## RESUMEN

**Objetivos:**

analizar la relación entre la espiritualidad y el cuidado humanizado en los profesionales de enfermería de un hospital chileno.

**Métodos:**

estudio observacional, analítico y transversal de abordaje cuantitativo realizado con 119 profesionales de enfermería de un hospital. Datos recopilados entre abril y julio 2024, mediante la aplicación de los cuestionarios: biosociodemográfico, Espiritualidad *SQ*, y *Nyberg’s Caring Assessment*. Para el análisis de datos se utilizó el programa SPSS versión 25.0.

**Resultados:**

se encontraron niveles medios de espiritualidad y cuidado humanizado, lo que refleja que las enfermeras(os) cuentan con competencias interpersonales para entregar cuidados holísticos, además, existe una correlación estadística significativa (p≤0,05) entre los constructos.

**Conclusiones:**

la espiritualidad determina el cuidado humanizado mediante un valor ético intrínseco, tal como señala la Teoría del Cuidado Humano de Jean Watson. Por ello, los hospitales deben considerar estos elementos en la formación y práctica de los profesionales de enfermería para entregar un cuidado holístico.

## INTRODUCTION

Spirituality is a dimension of the human being that has to do with caring for life^(1,2)^. In the context of health, this concept has its origins in palliative care when caring for people who do not respond to biomedical treatment^(3)^. Therefore, it is conceived as an essential dimension of the human being, recognized with intrinsic dignity, and which is related to the meaning of life, suffering, connection and transcendence^(4)^. This universal property of the person consists of the inexhaustible longing for plenitude and happiness that offers consolation and interventions in late stages of life, when death becomes conscious^(5-7)^. For the discipline of nursing, spirituality is a path that leads to the metaparadigm of care. This allows nurses to offer care permeated by love, hope, solidarity and active compassion and, thus, provide truly humane care that is recognizes, particularly for the person as a whole being and their spiritual dimension^(2,7,8)^. Through stimulation, this dimension enables brain activation^(9,10)^ and subsequent expression through emotions^(11)^, becoming a tool that allows the individual to feel and react according to their convictions^(12)^.

In the health context, humanized care was defined by the World Health Organization (WHO) as a process centered on people’s communication that is oriented towards transformation and essential spiritual understanding of life^(13)^. This process considers vulnerable people in their entirety, encompassing the social, physical, psychological and spiritual dimensions^(14)^. Thus, from a Levinasian perspective, it involves offering a warm welcome that receives the face of a subject with dignity and kindness from the perspective of a host^(15)^.

In her Philosophy and Theory of Human Caring, the author Jean Watson proposed a guide that illuminates the awareness of care based on 10 caritas factors or processes, which will allow us to understand the results obtained in this investigation^(7,8)^.

Thus, the relationship between spirituality and humanized care can be understood as a cyclical process for nursing, because if a nurse has training in spirituality, he or she will be able to be aware of separating “physiology”, which has value in itself, from what is “normal”. This has been applied, for example, in places such as Obstetric Nursing Units in Brazil with the Humanized Childbirth Model^(16,17)^. When nursing care focuses on people, health and the environment, based on discipline, nurses can offer care based on hope and love^(2,8)^. In this way, nursing care contributes to the relief of negative emotions and to the improvement of people’s quality of life, granting them autonomy and freedom, supported by scientific evidence to maintain safety in care and, thus, contribute positively to the humanization of health based on science, respect and social justice as part of ethical care^(16,18)^. In Chile, there is an empirical gap on this topic; therefore, providing new knowledge in this area allows us to respond to the comprehensive care that users of the health system require, contribute to new research and expand the field of action of this theoretical model. This study sought to answer the following research question: is there a relationship between spirituality and humanized care among nursing professionals in a Chilean hospital, in light of the Theory of Human Caring?

## OBJECTIVES

To analyze the relationship between spirituality and humanized care in nursing professionals in a Chilean hospital.

## METHODS

### Ethical aspects

Data collection followed the ethical principles of Ezequiel Emanuel^(19,20)^. The execution, dissemination and publication of the results were approved by the Scientific Ethics Committee of the Universidad Católica del Maule through Resolution No. 04/2024, and authorization was obtained from the Director of the health facility. In addition, free and informed consent was requested from all participants in this study.

### Theoretical-methodological framework

Jean Watson’s theory provides the assumptions as a theoretical framework for this research, in which humanized care includes the social, physical, psychological and spiritual dimensions of people^(7,8)^.

### Study design

To guide the methodology, the STROBE (Strengthening the Reporting of Observational Studies in Epidemiology) Statement^(21)^ was used. This is an observational, analytical, cross-sectional study with a quantitative approach^(22)^, carried out from April to June 2024 in a high-complexity hospital located in the VI region of Chile.

### Methodological procedures

#### Study setting

The study included 119 nursing professionals, from both genders, selected by non-probabilistic convenience sampling. This number is justified by theoretical-methodological criteria, being suitable for descriptive studies in specific clinical contexts^(22)^.

For data collection, Google Forms platform was used, through which an online self-report instrument was applied, lasting an average of 30 minutes, consisting of three sections: a bio-sociodemographic questionnaire, the Spirituality Questionnaire (SQ)^(23)^ and the Nyberg’s Caring Assessment Questionnaire^(24)^.

The Nyberg’s Caring Assessment Questionnaire, developed by Jan Nyberg in the United States, was adapted and validated for use in the Spanish language for the Chilean population^(24)^. This instrument allows the assessment of the characteristics of humanized care described in Jean Watson’s theory. It is a questionnaire with a 20-item Likert scale and five response options, scored from 1 to 5, with 1 being never, 2 occasionally, 3 sometimes, 4 frequently, and 5 always. The results allow the level of humanized care to be categorized into three ranges: low (67-73 points), medium (74-85 points), and high (86-98 points)^(25,26)^.

The Spirituality Questionnaire (SQ), also validated in Chile, consists of 29 items distributed across four dimensions: self-knowledge, beliefs, practices, and spiritual needs. Thus, spirituality is classified into three levels: high (116-102 points), medium (101-87 points) and low (86-29 points)^(23,27)^.

#### Participants selection

Participants were selected through convenience sampling. The approach protocol consisted of a promotional poster that allowed access to the Google Forms platform. This poster was distributed by the Sub directorate of Care Management (SDGC) via email, through a WhatsApp group of the Association of Nurses (ASENF) from the hospital itself, and also by the researcher in the services belonging to the hospital.

The inclusion criteria were: nursing professionals with a nursing diploma, with any work regime, contractual relationship and care, management and/or administrative work in services with direct care to users. The exclusion criteria were: nursing professionals on sick leave, legal vacation or administrative leave at the time of data collection.

Bias control: to reduce bias, all participants were included without discrimination by age, gender or other conditions; it is worth noting that the majority of professionals are women, which makes gender equality impossible. As for the instruments, they met validation and reliability criteria. Regarding data analysis, an anonymized database was used.

### Data analysis

The data were analyzed using descriptive statistics (mean and standard deviation) and the measure of association used was the Chi-square test of independence, with the respective 95% confidence interval and significance level lower than 5% (p < 0.05). The data were tabulated in Microsoft Office Excel and analyzed in the Statistical Package for the Social Sciences (SPSS) version 25.0.

## RESULTS

This study included 119 nursing professionals out of a total of 675 nurses from a Chilean hospital, with a higher proportion of female participants (85.7%), with an average age of 37 years old and a predominant age range between 34 and 43 years (40.3%). The minimum age recorded was 24 years old and the maximum was 74 years. Most participants identified with some religion (78.2%), with the Catholic religion predominating (62.2%). Most of them had between 1 and 5 years of professional experience (42.9%). Regarding continuing education, the most notable were short-term courses (37.0%) and advanced training courses (46.2%), with training in humanized care (27.7%) and spirituality (24.4%). The professionals belonged to 21 care units, the majority of which were from the Adult Medicine service (16.8%). Finally, regarding the type of care provided, the majority worked in inpatient services (87.4%) (Table 1).

**Table 1:** Bio-sociodemographic profile of nursing professionals in a hospital, Region VI, Chile (N=119)

Bio-sociodemographic variables (N=119)	ƒ	%
Gender		
Women	102	85.7%
Men	17	14.3%
Age (Average 37,34 , SD 7,908)		
24 to 33 years	47	39.5%
34 to 43 years	48	40.3%
44 to 53 years	21	17.6%
54 to 63 years	2	1.7%
64 to 74 years	1	0.8%
Do you identify with any religion?		
Yes	93	78.2%
No	26	21.8%
What religion do you identify with the most?		n=119
Catholic	74	62.2%
Christian	9	7.6%
Evangelical	9	7.6%
Methodist	1	0.8%
None	26	21.8%
Length of service in current position		
Less than 1 year	7	5.9%
1 to 5 years	51	42.9%
6 to 10 years	27	22.7%
11 to 20 years	39	32.8%
21 to 30 years	4	3.4%
31 to 40 years	1	0.8%
Contractual status		
Replacement	24	20.2%
Contractor (Fixed-term contract)	74	62.2%
Permanent (Indefinite term contract)	21	17.6%
Working hours		
Daytime	51	42.9%
Scale 12/12	68	57.1%
Continuous training		
Short course	44	37.0%
Improvement Course	55	46.2%
Postgraduate degree lato sensu	9	7.6%
Clinical Specialty/Residency Program	5	4.2%
Masters	6	5.0%
Training in humanized care		
Yes	33	27.7%
No	86	72.3%
Training in spirituality		
Yes	29	24.4%
No	90	75.6%
Service/unit/sector to which they belong		
Pain Relief	3	2.5%
Children's Surgery	2	1.7%
Adult Specialties and Procedures	8	6.7%
Hemodialysis	2	1.7%
Hemodynamics	2	1.7%
Home Hospitalization	7	5.9%
Adult Medicine	20	16.8%
Neurosurgery	7	5.9%
Oncology	2	1.7%
Surgical Center	7	5.9%
Pediatrics	2	1.7%
Traumatology	11	9.2%
PICHU Adults	6	5.0%
PICHU Child-Teenager	4	3.4%
CPU Adult	13	10.9%
CPU Child	6	5.0%
Emergency Adults	8	6.7%
Pediatric Emergency	3	2.5%
Adult ICU 3rd floor	2	1.7%
Adult ICU 5th floor	2	1.7%
Neonatal ICU	2	1.7%
Type of attention		
Outpatient care	15	12.6%
Hospital care	104	87.4%

*PICHU - Psychiatric Intensive Care Hospital Unit; CPU - Critical Patient Unit, UTI - Intensive Care Unit.*

Regarding humanized care, 71.4% presented an intermediate level (Table 2).

**Table 2:** Humanized care among nursing professionals in a hospital, Region VI, Chile (N=119)

Global level of attributes of humanized care	ƒ	%
High (86-98 pontos)	17	14.3%
Average (74-85 pontos)	85	71.4%
Low (67-73 pontos)	17	14.3%

Regarding spirituality, 43.7% had an average level (Table 3).

**Table 3:** Spirituality among nursing professionals in a hospital, Region VI, Chile (N=119)

Global Level of Spirituality	ƒ	%
High (102-116 pontos)	28	23.5%
Average (87-101 pontos)	52	43.7%
Low (29-86 pontos)	39	32.8%

In the relationship between spirituality and humanized care, a significant statistical association (p-value=0.049) was observed between both constructs.

Table 4 presents the results of the Chi-square test of independence, used to assess the association between spirituality and humanized care among nursing professionals at a Chilean hospital.

**Table 4:** Chi-square test of independence between bio-sociodemographic variables, spirituality, humanized care and their respective dimensions in nursing professionals of a hospital, VI Region, Chile (N=119)

Biosociodemographic Variables	Spirituality (gl)			Humanized care (gl)
	Spiritual beliefs	Spiritual practices	Spiritual needs	Phenomenological field
Gender	9.247^*^ ^*^ (p=0.010)	3.927 (p=0.140)	0.848 (p=0.655)	1.754 (p=0.416)
Has a religion	10.157^*^ ^*^ (p=0.006)	0.011 (p=0.994)	2.618 (p=0.270)	8.356^*^ (p=0.015)
Working hours	1.758 (p=0.415)	1.078 (p=0.583)	5.201 (p=0.074)	5.103 (p=0.078)
Training in spirituality	14.734^*^ ^*^ (p=0.001)	14.758^*^ ^*^ (p=0.001)	7.708^*^ (p=0.021)	2.438 (p=0.296)
Type of attention	1.574 (p=0.455)	1.860 (p=0.395)	3.404 (p=0.182)	7.149^*^ (p=0.028)

Statistical significance:

* p≤ 0.05;

** p≤ 0.01.

In the analysis of the relationship between the bio-sociodemographic profile and the dimensions of humanized care, it was identified that the phenomenological field dimension presents a significant association with religious identification (p=0.015) and with the type of care provided by nursing professionals - hospital or outpatient (p=0.028).

On the other hand, when analyzing the relationship between the bio-sociodemographic profile and the dimensions of spirituality, relevant associations were found. The spiritual beliefs dimension is significantly associated with gender (p=0.010), religious identification (p=0.006) and training in spirituality (p=0.001). Furthermore, the spiritual practices dimension is also related to training in spirituality (p=0.001), while the spiritual needs dimension presents a significant association with this same factor (p=0.021).

## DISCUSSION

The results of this research are discussed in light of Jean Watson’s Theory of Human Caring, a useful approach to addressing the spiritual dimension of the human being. In this theory, spirituality is presented as a protective element, both for the nurse who provides care and for the person receiving it^(8,17,28,29)^. The 10 caritas factors, which include sensitivity to human needs, the promotion of faith and hope, and respect for the dignity of others, constitute the basis for comprehensive and humanized care^(7,8)^. In this context, spirituality transcends the physical and emotional dimension, incorporating itself as an essential component to contribute to the formation of meaningful and humanizing relationships.

In the descriptive results, a high representation of female participants (85.7%) was observed, a trend historically present in nursing. This coincides with research in Ecuador and Indonesia, where female representation was also predominant, with 79% and 78.3%, respectively^(25,30)^. However, in recent decades, the discipline has evolved towards greater male inclusion, demonstrating its ability to perform care roles with professionalism and sensitivity^(31,32)^.

The average age of participants was 37 years, with a predominant range between 34 and 43 years, consistent with studies conducted in Brazil and Ecuador^(25,33)^. Furthermore, in this study, 78% of participants identified with some religion, with Catholicism being the predominant religion (62.2%). This data contrasts with the Encuesta Nacional Bicentenario UC 2021, in which only 42% declared themselves Catholic and 37% stated that they had no religion or were atheists^(34)^. These findings reflect a spiritual dimension present in nursing professionals, who, exposed to situations of suffering and stress, can find in religion or spirituality a source of emotional and psychological support^(28)^.

Regarding work experience, 42.9% of participants had between 1 and 5 years of experience, a result similar to that of Ecuador and Indonesia, where the majority of nurses had less than 5 years of experience, with 50% and 35.4%, respectively. This suggests that a considerable proportion of nurses are in the early stages of their professional development^(25,35)^.

With regard to contractual status, the renewable annual contract predominates (62.2%), followed by replacement (20.2%) and indefinite-term contracts (17.6%); in relation to working hours, the 12/12 shift is predominant (57.1%), while the daytime modality represents 42.9%. These results coincide with those found in 2017 in Chile^(36)^.

Regarding continuing education, the most important courses were advanced training (46.2%), followed by short-term courses (37%), while only 4.2% attended clinical specialty/residency programs. These results coincide with those found in Ecuador, where 79% had only a bachelor’s degree^(25)^. In terms of training in humanized care and spirituality, less than 30% of professionals received specific training in this area, which highlights the need to strengthen these skills^(28,33)^. In addition, the majority of participants worked in services such as Adult Medicine (16.8%) and Adult Critical Care Units (10.9%), and 87.4% provided services to hospitalized patients, a result comparable to the 80.7% found in Chile^(36)^.

### Attributes of humanized care and its dimensions

Regarding the observed results, the nurse-patient interaction dimension presented a high level of humanized care attributes (44.5%), which reveals that nursing professionals demonstrate deep respect for the patient’s needs, convey encouragement and hope, and remain sensitive to their demands. This finding reinforces the concept of humanized care, which highlights the importance of seeing the person as a whole being and not just as a disease or diagnosis, as explained in Jean Watson’s Theory of Human Caring^(7,8,24)^. In Argentina, it was found that 67.5% of nursing professionals in a Critical Patient Unit (CPU) expressed positive relevance regarding trust and respect for the other as a human being^(37)^. On the other hand, the transpersonal care relationship dimension presented an average level of attributes (65.5%), which highlights the importance of communicating an attitude of help and trust to others, setting aside time for care opportunities, maintaining a commitment to an ongoing relationship, believing that others have potential that can be achieved, focusing on helping others grow, listening carefully and being open to feedback from others, considering relationships before regulations, basing relationships on what is best for those involved, fully understanding the meaning of situations for people and going beyond the superficial to get to know them well^(7,8,24)^. In Argentina, it was found that 40.83% of nursing professionals always reflect these actions in their daily practice^(37)^.

The moment of care dimension presented an average level of humanized care attributes (64.7%), highlighting the importance of creativity in problem-solving and the application of technical skills in the care process. Although a technical focus is necessary, the results suggest that humanized care should not focus solely on techniques or procedures, but also on how these are implemented in a personalized way. A more integrated approach, including technical and interpersonal aspects, could further improve the results in this dimension^(7,8,38)^. In Argentina, it was found that 45.83% of nursing professionals frequently present these aspects of holistic care^(37)^.

Finally, the phenomenological field dimension, which obtained an average level of attributes (55.5%), reflects the awareness of the impact of spiritual forces on human care. This result highlights the importance of expressing positive and negative feelings, setting aside time for personal needs and growth, giving full consideration to situational factors, and understanding that spiritual forces contribute to human care^(8)^. Similarly, in Argentina, it was found that 37.5% of nursing professionals frequently present these aspects of holistic care^(37)^.

### Spirituality and its dimensions

Firstly, the self-knowledge dimension presented an average level (43.7%). Nursing professionals expressed satisfaction with themselves, a positive attitude towards themselves, confidence, compassion and the ability to extract positive learning from adverse situations. These findings are consistent with those reported in nursing students^(23,39)^, who also achieved an average level (41.3%), although they differed from the results found in students with Burnout, who presented a low level (93.5%)^(27)^.

On the other hand, the spiritual beliefs dimension also presented an average level (40.3%). Nurses indicated that their spirituality influences the definition of their goals, their self-identity and their integral approach to life, being integrated into their daily lives. As in the previous dimension, these results coincide with those found in Chile^(23,39)^, where nursing students obtained an average level (41.3%), but contrast with another study, which found a low level (75.9%)^(27)^.

As for the spiritual practices dimension, a high level was identified (42.0%). Professionals reported participating in activities such as reading about spirituality, meditation, prayer, contact with nature, and using silence to reflect and connect with themselves^(23)^. These results differ from other studies conducted in Chile, which identified a medium level (41.3%)^(39)^, and from another study that found a low level (53.8%)^(27)^.

Finally, the spiritual needs dimension reached a low level (52.1%). Participants expressed seeking a purpose in life, enjoying music, maintaining meaningful interpersonal relationships, and developing a full life, among other needs. This result differs from others conducted in Chile^(39)^, which reported a high level (38.0%), and in which the low level predominated significantly (97.5%)^(27)^.

### Relationship between spirituality and humanized care

This study identified a statistically significant association between spirituality and humanized care (p = 0.049), which suggests that there is a relationship between both constructs. However, since the p value is very close to the conventional significance threshold (0.05), this is a marginal association; therefore, the results should be interpreted with caution and considered within the clinical and methodological context of the study.

This result is consistent with previous research conducted in Peru^(40)^. While nursing students tend to have greater spirituality, due to their training and a more idealistic perspective^(39)^, professionals exhibit average levels of humanized care, probably influenced by work stress^(41,42)^. This finding highlights the lack of integration of spirituality as an essential component of humanized care^(28,41)^.

The phenomenological dimension of humanized care favors the spiritual connection between professionals and patients^(7,8)^, highlighting the importance of spiritual beliefs and practices to provide comfort and reduce pain^(43)^.

Training in spirituality is essential to overcome barriers and provide adequate support, especially in palliative care^(44)^. Furthermore, spiritual beliefs, influenced by religious factors and gender, affect the perception of humanized care^(45)^.

Hospital care allows for a greater perception of humanized care compared to outpatient care, due to the prolonged interaction between nurses and patients^(46)^. The results reinforce that greater training in spirituality increases its levels (p = 0.003), promoting a more authentic and humanizing nursing practice^(47)^.

All of this reflects, in light of Jean Watson’s theory, that the nursing professionals participating in this study have interpersonal skills to provide humanized care; however, there is an urgent need to recognize one’s own subjectivity, promote human dignity and understand the spiritual dimension in order to recognize the individuality of each person.

### Study limitations

Among the limitations identified are, firstly, the use of convenience sampling, since participants were not selected randomly. This, combined with the fact that the nurses work in a single hospital, although they come from different units, limits the possibility of generalizing the results to other health centers, whether public or private. In addition, the low voluntary participation and the limited previous application of the instruments in Chile and in the Latin American context represent additional challenges that may influence the interpretation. Added to this is the limitation of the analysis by gender, given that the majority of professionals are women.

### Contribution to the areas of nursing, health and public health

This study included the participation of nursing professionals working in a highly complex institution, in which average levels of spirituality and humanized care were identified, impacting the care provided by the category to the population. This information provides a knowledge base for future interventions in the area. Understanding the relationship between spirituality and humanized care highlights the importance of integrating these aspects into the training and practice of nursing professionals, promoting an approach of comprehensive and holistic care that meets the physical, emotional and spiritual needs of the people under their care. It also signals the need to incorporate this theme into the training of future nurses.

## CONCLUSIONS

The results of this research confirm a significant relationship between spirituality and humanized care in the practice of nursing professionals, highlighting that the spiritual dimension is deeply linked to the metaparadigm of nursing, which integrates person, environment, health and care. This link highlights the need to consider spirituality as an essential component of humanized care, based on ethical values such as empathy and respect for human dignity.

This highlights the importance of incorporating comprehensive training into undergraduate nursing programs, ensuring that the dimension of spirituality is considered an essential element in the preparation of future health professionals. In this way, they will be qualified and familiar with the subject to address this dimension in their clinical practice. In this context, Jean Watson’s Theory of Human Caring presents itself as a solid theoretical framework that guides the integration of spirituality into humanized care, strengthening the quality and depth of the care provided.

As a recommendation from the research team, it is suggested to expand the analysis through qualitative research, so that nursing professionals can express their own experiences or meanings regarding humanized care and spirituality when providing care, both in the hospital and outpatient contexts. It is also recommended that future studies consider the analysis of health institutions in promoting environments that value the spiritual dimension of nursing professionals.

## Data Availability

The research data are available within the article.
