# Supplementary material for: Relationship between spirituality and humanized care in nursing professionals at a Chilean hospital
Source: Rev Bras Enferm. 2025 Dec 15;78(5):e20240598. doi: 10.1590/0034-7167-2024-0598 (PMC12707853; doi:10.1590/0034-7167-2024-0598)
Supplement: Translation

# Translation: Relação entre espiritualidade e cuidado humanizado em profissionais de enfermagem de um hospital chileno

**DOI:** 10.1590/0034-7167-2024-0598pt

**Authors:** Daniela Jacqueline Espinoza-Padilla, Margarita del Carmen Poblete-Troncoso, Verónica Teresa Guerra-Guerrero, Cláudia Zamberlan, Dirce Stein Backes

**Affiliations:** I Hospital Dr. Franco Ravera Zunino. Rancagua, Chile; II Universidad Católica del Maule, Centro de Investigación del Cuidado. Talca, Chile; III Universidade Franciscana. Santa Maria, Rio Grande do Sul, Brasil

**Keywords:** Espiritualidade, Humanização do Cuidado, Enfermagem, Cuidados de Enfermagem, Hospitais Públicos.

## Abstract

**Objetivos:**

analisar a relação entre a espiritualidade e o cuidado humanizado em profissionais de enfermagem de um hospital chileno.

**Métodos:**

estudo observacional, analítico e transversal de abordagem quantitativa realizado com 119 profissionais de enfermagem de um hospital. Os dados foram coletados entre abril e julho de 2024, mediante a aplicação dos questionários: biossociodemográfico, Espiritualidade *SQ* e *Nyberg’s Caring Assessment*. Para a análise dos dados utilizou-se o programa SPSS versão 25.0.

**Resultados:**

encontraram-se níveis médios de espiritualidade e cuidado humanizado, o que reflete que as enfermeiras(os) possuem competências interpessoais para oferecer cuidados holísticos; além disso, existe uma correlação estatística significativa (p≤0,05) entre os constructos.

**Conclusões:**

a espiritualidade determina o cuidado humanizado por meio de um valor ético intrínseco, conforme assinala a Teoria do Cuidado Humano de Jean Watson. Portanto, os hospitais devem considerar esses elementos na formação e na prática dos profissionais de enfermagem para fornecer um cuidado holístico.

## INTRODUÇÃO

A espiritualidade é uma dimensão do ser humano orientada ao cuidado da vida^(1,2)^. No âmbito da saúde, esse conceito tem sua origem nos cuidados paliativos ao atender pessoas que não respondem ao tratamento biomédico^(3)^. Portanto, concebe-se como uma dimensão essencial do ser humano, reconhecido com dignidade intrínseca, e que está relacionada ao sentido da vida, do sofrimento, da conexão e da transcendência^(4)^. Essa propriedade universal da pessoa consiste no anseio inesgotável de plenitude e felicidade que oferece consolo e intervenções em fases tardias da vida, quando a morte se torna consciente^(5-7)^.

Para a disciplina de enfermagem, a espiritualidade é um caminho que conduz ao metaparadigma do cuidado. Isso permite às enfermeiras e enfermeiros oferecer uma atenção permeada de amor, esperança, solidariedade e compaixão ativa e, assim, conceder cuidados verdadeiramente humanos, nos quais se reconheça a integralidade da pessoa e, em especial, sua dimensão espiritual^(2,7,8)^. Por meio de um estímulo, essa dimensão possibilita a ativação cerebral^(9,10)^ e a posterior manifestação por meio das emoções^(11)^, tornando-se uma ferramenta que permite ao indivíduo sentir e reagir de acordo com suas convicções^(12)^.

O cuidado humanizado, no contexto sanitário, foi definido pela Organização Mundial da Saúde (OMS) como um processo centrado na comunicação das pessoas que se orienta para a transformação e compreensão espiritual essencial da vida^(13)^. Esse processo considera as pessoas vulneráveis em sua globalidade, abrangendo as dimensões social, física, psicológica e espiritual^(14)^. Assim, sob a ótica levinasiana, implica oferecer uma boa acolhida que receba o rosto de um sujeito com dignidade e bondade a partir da perspectiva de um anfitrião^(15)^.

A autora Jean Watson propôs, em sua Filosofia e Teoria do Cuidado Humano, um guia que ilumina a consciência do cuidado baseado em 10 fatores ou processos caritas, o que permitirá compreender os resultados obtidos nesta investigação^(7,8)^.

Assim, a relação entre a espiritualidade e o cuidado humanizado pode ser compreendida como um processo cíclico para a enfermagem, pois, se uma enfermeira ou enfermeiro possui formação em espiritualidade, poderá ter a consciência de separar a “fisiologia”, que possui valor em si mesma, do que é “normal”. Isso vem sendo aplicado, por exemplo, em locais como as Unidades de Enfermagem Obstétrica no Brasil com o Modelo de Parto Humanizado^(16,17)^. Quando a atenção de enfermagem se concentra nas pessoas, na saúde e no ambiente, com base na disciplina, as enfermeiras e os enfermeiros podem oferecer um cuidado fundamentado na esperança e no amor^(2,8)^. Dessa forma, os cuidados de enfermagem contribuem para o alívio das emoções negativas e para a melhora da qualidade de vida das pessoas, concedendo-lhes autonomia e liberdade, respaldadas pela evidência científica para manter a segurança na atenção e, assim, contribuir positivamente para a humanização da saúde baseada na ciência, no respeito e na justiça social como parte do cuidado ético^(16,18)^.

No Chile, existe uma lacuna empírica sobre essa temática; assim, fornecer novos conhecimentos nessa área permite responder ao cuidado integral que os usuários do sistema de saúde requerem, contribuir para novas investigações e ampliar o campo de ação desse modelo teórico. Este estudo buscou responder à seguinte pergunta de pesquisa: existe relação entre a espiritualidade e o cuidado humanizado nos profissionais de enfermagem de um hospital chileno, à luz da Teoria do Cuidado Humano?

## OBJETIVOS

Analisar a relação entre a espiritualidade e o cuidado humanizado em profissionais de enfermagem de um hospital chileno.

## MÉTODOS

### Aspectos éticos

A coleta de dados seguiu os princípios éticos de Ezequiel Emanuel^(19,20)^. A execução, difusão e divulgação dos resultados foi aprovada pelo Comitê Ético Científico da *Universidad Católica del Maule* por meio da Resolução n.º 04/2024, e foi obtida a autorização do Diretor do estabelecimento de saúde. Além disso, solicitou-se um consentimento livre e esclarecido a todos os participantes deste estudo.

### Referencial teórico-metodológico

A teoria de Jean Watson fornece os pressupostos como referencial teórico desta investigação, na qual o cuidado humanizado inclui a dimensão social, física, psicológica e espiritual das pessoas^(7,8)^.

### Desenho do estudo

Para orientar a metodologia, utilizou-se a Declaração STROBE (*Strengthening the Reporting of Observational Studies in Epidemiology*)^(21)^. Trata-se de um estudo observacional, analítico e transversal com abordagem quantitativa^(22)^, realizado de abril a junho de 2024 em um hospital de alta complexidade, localizado na VI região, no Chile.

### Procedimentos metodológicos

#### Cenário do estudo

O estudo incluiu 119 profissionais de enfermagem, enfermeiras e enfermeiros, selecionados por amostragem não probabilística por conveniência. Esse quantitativo justifica-se por critérios teórico-metodológicos, sendo adequado para estudos descritivos em contextos clínicos específicos^(22)^.

Para a coleta de dados, utilizou-se a plataforma *Google Forms*, por meio da qual foi aplicado um instrumento on-line de autorrelato, com duração de 30 minutos, composto por três seções: um questionário biossociodemográfico, o *Cuestionario de Espiritualidad* (*SQ*)^(23)^ e o Questionário *Nyberg’s Caring Assessment* ^(24)^.

O Questionáro *Nyberg’s Caring Assessment*, desenvolvido por Jan Nyberg nos Estados Unidos, foi adaptado e validado para o idioma espanhol na população chilena^(24)^. Esse instrumento permite avaliar as características do cuidado humanizado descritas na teoria de Jean Watson. Trata-se de um questionário com escala Likert de 20 itens e cinco opções de resposta, pontuadas de 1 a 5, correspondendo 1 a nunca, 2 ocasionalmente, 3 às vezes, 4 frequentemente e 5 sempre. Os resultados possibilitam categorizar o nível de cuidado humanizado em três faixas: baixo (67-73 pontos), médio (74-85 pontos) e alto (86-98 pontos)^(25,26)^.

O *Cuestionario de Espiritualidad* (*SQ*), também validado no Chile, é composto por 29 itens distribuídos em quatro dimensões: autoconhecimento, crenças, práticas e necessidades espirituais. Dessa forma, a espiritualidade classifica-se em três níveis: alta (116-102 pontos), média (101-87 pontos) e baixa (86-29 pontos)^(23,27)^.

### Seleção de participantes

Os participantes foram selecionados por meio de amostragem por conveniência. O protocolo de aproximação consistiu em um cartaz de divulgação que permitia o acesso à plataforma *Google Forms.* Esse cartaz foi difundido pela Subdireção de Gestão do Cuidado (SDGC) via e-mail, por um grupo de *WhatsApp* da Associação de Enfermeiras e Enfermeiros (ASENF) do próprio hospital e, também, pela pesquisadora nos serviços pertencentes ao hospital.

Os critérios de inclusão foram: profissionais de enfermagem com diploma de enfermeira(o), qualquer regime de trabalho, vínculo contratual e atuação assistencial, de gestão e/ou administrativa em serviços com atendimento direto aos usuários. Os critérios de exclusão foram: profissionais de enfermagem em licença médica, férias legais ou licenças administrativas no momento da coleta de dados.

Controle de vieses: para reduzir vieses, incluíram-se todos os participantes sem discriminar por idade, gênero ou outras condições; cabe ressaltar que a maioria dos profissionais é composta por mulheres, o que impossibilita a igualdade de gênero. Quanto aos instrumentos, estes atenderam a critérios de validação e confiabilidade. No que se refere à análise de dados, utilizou-se uma base de dados anonimizada.

Análise de dados

Os dados foram analisados com estatística descritiva (média e desvio-padrão) e a medida de associação utilizada foi o Teste de independência do Qui-quadrado, com o respectivo intervalo de confiança de 95% e nível de significância inferior a 5% (p < 0,05). Os dados foram tabulados no *Microsoft Office Excel* e analisados no Pacote Estatístico para Ciências Sociais (SPSS) versão 25.0.

## RESULTADOS

Este estudo incluiu 119 profissionais de enfermagem de um total de 675 enfermeiras(os) de um hospital chileno, observando-se maior proporção de participantes do sexo feminino (85,7%), com idade média de 37 anos e faixa etária predominante entre 34 e 43 anos (40,3%). A idade mínima registrada foi de 24 anos e a máxima de 74 anos. A maioria dos participantes identificou-se com alguma religião (78,2%), predominando a religião católica (62,2%). A maior parte possuía entre 1 e 5 anos de experiência profissional (42,9%).

Quanto à formação continuada, destacaram-se a realização de cursos de curta duração (37,0%) e cursos de aperfeiçoamento (46,2%), com formação em cuidado humanizado (27,7%) e espiritualidade (24,4%). Os profissionais pertenciam a 21 unidades de atendimento, sendo a maioria do serviço de Medicina de Adultos (16,8%). Por fim, quanto ao tipo de atendimento prestado, a maioria atuava em serviços de internação (87,4%) (Tabela 1).

**Tabela 1:** Perfil biossociodemográfico dos profissionais de enfermagem de um hospital, VI Região, Chile (N=119)

Variáveis biossociodemográficas (N=119)	ƒ	%
Sexo		
Mulher	102	85,7%
Homem	17	14,3%
Idade (Média 37,34 , DP 7,908)		
24 a 33 anos	47	39,5%
34 a 43 anos	48	40,3%
44 a 53 anos	21	17,6%
54 a 63 anos	2	1,7%
64 a 74 anos	1	0,8%
Se identifica com alguma religião		
Sim	93	78,2%
Não	26	21,8%
Qual é a religião que o identifica?		n=119
Católica	74	62,2%
Cristã	9	7,6%
Evangélica	9	7,6%
Metodista	1	0,8%
Nenhuma	26	21,8%
Tempo de serviço no cargo atual		
Menos de 1 ano	7	5,9%
1 a 5 anos	51	42,9%
6 a 10 anos	27	22,7%
11 a 20 anos	39	32,8%
21 a 30 anos	4	3,4%
31 a 40 anos	1	0,8%
Situação contratual		
Substituição	24	20,2%
Contratado (Contrato por prazo determinado)	74	62,2%
Efetivo (Contrato por tempo indeterminado)	21	17,6%
Jornada de trabalho		
Diurno	51	42,9%
Escala 12/12	68	57,1%
Formação contínua		
Curso de curta duração	44	37,0%
Curso de Aperfeiçoamento	55	46,2%
Pós-graduação lato senso	9	7,6%
Programa de especialidade clínica/residência	5	4,2%
Mestrado	6	5,0%
Formação em cuidado humanizado		
Sim	33	27,7%
Não	86	72,3%
Formação em espiritualidade		
Sim	29	24,4%
Não	90	75,6%
Serviço/unidade/setor ao que pertence		
Alívio da Dor	3	2,5%
Cirurgia Infantil	2	1,7%
Especialidades e Procedimentos Adulto	8	6,7%
Hemodiálise	2	1,7%
Hemodinâmica	2	1,7%
Hospitalização Domiciliar	7	5,9%
Medicina do Adulto	20	16,8%
Neurocirurgia	7	5,9%
Oncologia	2	1,7%
Centro Cirúrgico	7	5,9%
Pediatria	2	1,7%
Traumatologia	11	9,2%
UHCIP Adultos	6	5,0%
UHCIP Infanto-Adolescente	4	3,4%
UPC Adulto	13	10,9%
UPC Infantil	6	5,0%
Urgência Adultos	8	6,7%
Urgência Pediátrica	3	2,5%
UTI Adulto 3° andar	2	1,7%
UTI Adulto 5° andar	2	1,7%
UTI Neonatal	2	1,7%
Tipo de atenção		
Atenção ambulatorial	15	12,6%
Atenção hospitalar	104	87,4%

*UHCIP - Unidade Hospitalar de Cuidados Intensivos de Psiquiatria; UPC - Unidade de Paciente Crítico, UTI - Unidade de Terapia Intensiva.*

Em relação ao cuidado humanizado, 71,4% apresentaram, nível médio (Tabela 2).

**Tabela 2:** Cuidado humanizado nos profissionais de enfermagem de um hospital, VI Região, Chile (N=119)

Nível global de atributos do cuidado humanizado	ƒ	%
Alto (86-98 pontos)	17	14,3%
Médio (74-85 pontos)	85	71,4%
Baixo (67-73 pontos)	17	14,3%

Em relação à espiritualidade, 43,7% apresentaram nível médio (Tabela 3).

**Tabela 3:** Espiritualidade nos profissionais de enfermagem de um hospital, VI Região, Chile (N=119)

Nível global de Espiritualidade	ƒ	%
Alto (102-116 pontos)	28	23,5%
Médio (87-101 pontos)	52	43,7%
Baixo (29-86 pontos)	39	32,8%

Na relação entre a espiritualidade e o cuidado humanizado, observou-se uma associação estatística significativa (p-valor=0,049) entre ambos os constructos.

A Tabela 4 apresenta os resultados do teste de independência do Qui-quadrado, utilizado para avaliar a associação entre a espiritualidade e o cuidado humanizado nos profissionais de enfermagem de um hospital chileno.

**Tabela 4:** Teste de independência do Qui-quadrado entre as variáveis biossociodemográficas, a espiritualidade, o cuidado humanizado e suas respectivas dimensões nos profissionais de enfermagem de um hospital, VI Região, Chile (N=119)

Variáveis biossociodemográficas	Espiritualidade (gl)			Cuidado humanizado (gl)
	Crenças espirituais	Práticas espirituais	Necessidades espirituais	Campo fenomenológico
Sexo	9,247^*^ ^*^ (p=0,010)	3,927 (p=0,140)	0,848 (p=0,655)	1,754 (p=0,416)
Se identifica com alguma religião	10,157^*^ ^*^ (p=0,006)	0,011 (p=0,994)	2,618 (p=0,270)	8,356^*^ (p=0,015)
Jornada de trabalho	1,758 (p=0,415)	1,078 (p=0,583)	5,201 (p=0,074)	5,103 (p=0,078)
Formação em espiritualidade	14,734^*^ ^*^ (p=0,001)	14,758^*^ ^*^ (p=0,001)	7,708^*^ (p=0,021)	2,438 (p=0,296)
Tipo de atenção	1,574 (p=0,455)	1,860 (p=0,395)	3,404 (p=0,182)	7,149^*^ (p=0,028)

Significância estatística:

* p≤ 0,05;

** p≤ 0,01.

Na análise da relação entre o perfil biossociodemográfico e as dimensões do cuidado humanizado, identificou-se que a dimensão campo fenomenológico apresenta uma associação significativa com a identificação religiosa (p=0,015) e com o tipo de atenção prestada pelos profissionais de enfermagem - hospitalar ou ambulatorial (p=0,028).

Por outro lado, ao analisar a relação entre o perfil biossociodemográfico e as dimensões da espiritualidade, foram encontradas associações relevantes. A dimensão crenças espirituais está significativamente associada ao sexo (p=0,010), à identificação religiosa (p=0,006) e à formação em espiritualidade (p=0,001). Além disso, a dimensão práticas espirituais também se relaciona com a formação em espiritualidade (p=0,001), enquanto a dimensão necessidades espirituais apresenta uma associação significativa com esse mesmo fator (p=0,021).

## DISCUSSÃO

Os resultados desta investigação são discutidos à luz da Teoria do Cuidado Humano de Jean Watson, um enfoque útil para abordar a dimensão espiritual do ser humano. Nessa teoria, a espiritualidade apresenta-se como elemento protetor, tanto para a enfermeira(o) que presta cuidados quanto para a pessoa que os recebe^(8,17,28,29)^. Os 10 fatores caritas, que incluem a sensibilidade às necessidades humanas, a promoção da fé e da esperança e o respeito pela dignidade do outro, constituem a base para um cuidado integral e humanizado^(7,8)^. Nesse contexto, a espiritualidade transcende a dimensão física e emocional, incorporando-se como componente essencial para contribuir à formação de relações significativas e humanizadoras.

Nos resultados descritivos, observou-se elevada representação de participantes do sexo feminino (85,7%), tendência historicamente presente na enfermagem. Isso coincide com investigações no Equador e na Indonésia, onde a representação feminina também foi predominante, com 79% e 78,3%, respectivamente^(25,30)^. Contudo, nas últimas décadas a disciplina evoluiu para maior inclusão masculina, evidenciando sua capacidade de desempenhar papeis de cuidado com profissionalismo e sensibilidade^(31,32)^.

A idade média dos participantes foi de 37 anos, com faixa predominante entre 34 e 43 anos, consistente com estudos realizados no Brasil e no Equador^(25,33)^. Além disso, neste estudo, 78% dos participantes identificaram-se com alguma religião, sendo a Católica a predominante (62,2%). Esse dado contrasta com a *Encuesta Nacional Bicentenario* UC 2021, na qual apenas 42% se declararam católicos e 37% afirmaram não ter religião ou serem ateus^(34)^. Esses achados refletem uma dimensão espiritual presente nos profissionais de enfermagem, que, expostos a situações de sofrimento e estresse, podem encontrar na religião ou na espiritualidade uma fonte de apoio emocional e psicológico^(28)^.

Quanto à experiência laboral, 42,9% dos participantes possuem entre 1 e 5 anos de antiguidade, resultado semelhante ao de Equador e Indonésia, onde a maioria das enfermeiras(os) tem menos de 5 anos de experiência, com 50% e 35,4%, respectivamente. Isso sugere que uma proporção considerável de enfermeiras(os) se encontra nas primeiras etapas de seu desenvolvimento profissional^(25,35)^.

No que se refere à situação contratual, predomina a modalidade de contrato anual renovável (62,2%), seguida de substituição (20,2%) e contrato por tempo indeterminado (17,6%); em relação à jornada de trabalho, a escala 12/12 é predominante (57,1%), enquanto a modalidade diurna representa 42,9%. Esses resultados coincidem com os encontrados em 2017 no Chile^(36)^.

Quanto à formação continuada, destacam-se os cursos de aperfeiçoamento (46,2%), seguidos por cursos de curta duração (37%), enquanto apenas 4,2% cursaram programas de especialidade clínica/residência. Esses resultados coincidem com os encontrados no Equador, onde 79% possuíam apenas o grau de licenciatura^(25)^. Em termos de formação em cuidado humanizado e espiritualidade, menos de 30% dos profissionais receberam capacitação específica nessa área, o que ressalta a necessidade de fortalecer essas competências^(28,33)^. Além disso, a maioria dos participantes trabalhava em serviços como Medicina de Adulto (16,8%) e Unidade de Paciente Crítico Adulto (10,9%), e 87,4% prestavam serviços a pacientes hospitalizados, resultado comparável aos 80,7% encontrados no Chile^(36)^.

### Atributos do cuidado humanizado e suas dimensões

Com relação aos resultados observados, a dimensão interação enfermeira-paciente apresentou um nível alto de atributos do cuidado humanizado (44,5%), o que revela que os profissionais de enfermagem demonstram profundo respeito pelas necessidades do paciente, transmitem incentivo e esperança e permanecem sensíveis às suas demandas. Esse achado reforça o conceito de cuidado humanizado, que destaca a importância de ver a pessoa como um ser integral e não apenas como uma doença ou diagnóstico, conforme exposto na Teoria do Cuidado Humano de Jean Watson^(7,8,24)^. Na Argentina, verificou-se que 67,5% dos profissionais de enfermagem de uma Unidade de Paciente Crítico (UPC) manifestaram relevância positiva quanto à confiança e ao respeito pelo outro como ser humano^(37)^.

Por outro lado, a dimensão relação de cuidado transpessoal apresentou nível médio de atributos (65,5%), o que ressalta a importância de comunicar aos outros uma atitude de ajuda e confiança, reservar tempo para oportunidades de cuidado, manter compromisso com uma relação contínua, acreditar que os outros têm um potencial que pode ser alcançado, concentrar-se em ajudar o crescimento alheio, escutar atentamente e estar aberto ao *feedback* das pessoas, considerar as relações antes dos regulamentos, basear os vínculos no que é melhor para os envolvidos, compreender plenamente o significado das situações para as pessoas e ir além do superficial para conhecê-las bem^(7,8,24)^. Na Argentina, encontrou-se que 40,83% dos profissionais de enfermagem sempre refletem essas ações em sua prática diária^(37)^.

A dimensão momento de cuidado apresentou nível médio de atributos do cuidado humanizado (64,7%), salientando a importância da criatividade na solução de problemas e da aplicação de habilidades técnicas no processo assistencial. Embora o enfoque técnico seja necessário, os resultados sugerem que o cuidado humanizado não deve centrar-se apenas em técnicas ou procedimentos, mas também em como estes são implementados de forma personalizada. Um enfoque mais integrado, que inclua aspectos técnicos e interpessoais, poderia melhorar ainda mais os resultados nessa dimensão^(7,8,38)^. Na Argentina, identificou-se que 45,83% dos profissionais de enfermagem frequentemente apresentam esses aspectos do cuidado holístico^(37)^.

Por fim, a dimensão campo fenomenológico, que obteve nível médio de atributos (55,5%), reflete a consciência do impacto das forças espirituais no cuidado humano. Esse resultado evidencia a importância de expressar sentimentos positivos e negativos, reservar tempo para necessidades pessoais e crescimento, conceder plena consideração aos fatores situacionais e compreender que as forças espirituais contribuem para o cuidado humano^(8)^. Do mesmo modo, na Argentina, verificou-se que 37,5% dos profissionais de enfermagem frequentemente apresentam esses aspectos do cuidado holístico^(37)^.

### Espiritualidade e suas dimensões

Primeiramente, a dimensão autoconhecimento apresentou nível médio (43,7%). Os profissionais de enfermagem manifestaram satisfação consigo mesmos, atitude positiva em relação a si, confiança, compaixão e capacidade de extrair aprendizados positivos de situações adversas. Esses achados são consistentes com os relatados em estudantes de enfermagem^(23,39)^, que também alcançaram nível médio (41,3%), embora diferiram dos resultados encontrados em estudantes com Burnout, que apresentam nível baixo (93,5%)^(27)^.

Por outro lado, a dimensão crenças espirituais também apresentou nível médio (40,3%). As enfermeiras(os) assinalaram que sua espiritualidade influencia a definição de suas metas, sua autoidentidade e sua abordagem integral de vida, estando integrada em seu cotidiano. Assim como na dimensão anterior, esses resultados coincidem com os encontrados no Chile^(23,39)^, onde estudantes de enfermagem obtiveram nível médio (41,3%), mas contrastam com outro estudo, que encontrou nível baixo (75,9%)^(27)^.

Quanto à dimensão práticas espirituais, identificou-se nível alto (42,0%). Os profissionais relataram participação em atividades como leitura sobre espiritualidade, meditação, oração, contato com a natureza e uso do silêncio para refletir e conectar-se consigo mesmos^(23)^. Esses resultados diferem de outros estudos realizados no Chile, nos quais foi identificado nível médio (41,3%)^(39)^, e de outro estudo que encontrou nível baixo (53,8%)^(27)^.

Por fim, a dimensão necessidades espirituais alcançou nível baixo (52,1%). Os participantes expressaram buscar um propósito na vida, apreciar música, manter relações interpessoais significativas e desenvolver uma vida plena, entre outras necessidades. Esse resultado difere de outros realizados no Chile^(39)^, que reportaram nível alto (38,0%), e nos quais o nível baixo predominou significativamente (97,5%)^(27)^.

### Relação entre a espiritualidade e o cuidado humanizado

Nesta investigação identificou-se uma associação estatisticamente significativa entre a espiritualidade e o cuidado humanizado (p = 0,049), o que sugere existir relação entre ambos os constructos. Contudo, em razão do valor de *p* situar-se muito próximo do limiar convencional de significância (0,05), trata-se de uma associação marginal; assim, os resultados devem ser interpretados com cautela e considerados dentro do contexto clínico e metodológico do estudo.

Esse resultado é consistente com pesquisa anterior realizada no Peru^(40)^. Enquanto estudantes de enfermagem tendem a apresentar maior espiritualidade, em virtude da formação e de uma perspectiva mais idealista^(39)^, os profissionais exibem níveis médios de cuidado humanizado, provavelmente influenciados pelo estresse laboral^(41,42)^. Tal achado evidencia a falta de integração da espiritualidade como componente essencial do cuidado humanizado^(28,41)^.

A dimensão fenomenológica do cuidado humanizado favorece a conexão espiritual entre profissionais e pacientes^(7,8)^, destacando a importância das crenças e práticas espirituais para proporcionar consolo e reduzir a dor^(43)^.

A formação em espiritualidade é fundamental para superar barreiras e oferecer acompanhamento adequado, especialmente em cuidados paliativos^(44)^. Além disso, as crenças espirituais, influenciadas por fatores religiosos e pelo sexo, afetam a percepção do cuidado humanizado^(45)^.

A atenção hospitalar permite maior percepção de cuidado humanizado em comparação com a atenção ambulatorial, em razão da interação prolongada entre enfermeiros e pacientes^(46)^. Os resultados reforçam que maior formação em espiritualidade eleva seus níveis (p = 0,003), promovendo uma prática de enfermagem mais autêntica e humanizadora^(47)^.

Tudo isso reflete, à luz da teoria de Jean Watson, que os profissionais de enfermagem participantes deste estudo possuem competências interpessoais para oferecer cuidado humanizado; contudo, há necessidade premente de reconhecer a própria subjetividade, promover a dignidade humana e compreender a dimensão espiritual a fim de reconhecer a individualidade de cada pessoa.

### Limitações do estudo

Entre as limitações identificadas estão, em primeiro lugar, o uso de amostragem por conveniência, pois os participantes não foram selecionados aleatoriamente. Isso, somado ao fato de que as enfermeiras e os enfermeiros trabalham em um único hospital, embora provenientes de diversas unidades, restringe a possibilidade de generalizar os resultados a outros centros de saúde, públicos ou privados. Além disso, a baixa adesão voluntária e a limitada aplicação prévia dos instrumentos no Chile e no contexto latino-americano representam desafios adicionais que podem influenciar a interpretação. Soma-se a isso a limitação da análise por gênero, dado que a maioria dos profissionais é composta por mulheres.

### Contribuição para as áreas de enfermagem, saúde e saúde pública

Este estudo contou com a participação de profissionais de enfermagem que atuam em instituição de alta complexidade, na qual se identificaram níveis médios de espiritualidade e de cuidado humanizado, impactando a atenção prestada pela categoria à população. Essas informações oferecem uma base de conhecimento para futuras intervenções na área. A compreensão da relação entre espiritualidade e cuidado humanizado evidencia a importância de integrar esses aspectos à formação e à prática dos profissionais de enfermagem, promovendo uma abordagem de cuidado integral e holístico que atenda às necessidades físicas, emocionais e espirituais das pessoas sob sua responsabilidade. Também sinaliza a necessidade de incorporar essa temática na formação dos futuros enfermeiros.

## CONCLUSÕES

Os resultados desta investigação confirmam uma relação significativa entre a espiritualidade e o cuidado humanizado na prática dos profissionais de enfermagem, destacando que a dimensão espiritual está profundamente vinculada ao metaparadigma da enfermagem, que integra pessoa, ambiente, saúde e cuidado. Esse vínculo ressalta a necessidade de considerar a espiritualidade como componente essencial do cuidado humanizado, fundamentado em valores éticos como empatia e respeito pela dignidade humana.

Isso evidencia a importância de incorporar uma formação integral nos programas de graduação em enfermagem, garantindo que a dimensão da espiritualidade seja considerada elemento essencial na preparação dos futuros profissionais de saúde. Dessa forma, estarão capacitados e familiarizados com a temática para abordar essa dimensão em sua prática clínica. Nesse contexto, a Teoria do Cuidado Humano de Jean Watson apresenta-se como um marco teórico sólido que orienta a integração da espiritualidade no cuidado humanizado, fortalecendo a qualidade e a profundidade da atenção prestada.

Como recomendação da equipe investigadora, sugere-se ampliar a análise mediante investigação de abordagem qualitativa, para que os profissionais de enfermagem possam expressar suas próprias vivências ou significados referentes ao cuidado humanizado e à espiritualidade ao prestar cuidados, tanto no contexto hospitalar quanto no ambulatorial. Recomenda-se ainda que estudos futuros considerem a análise das instituições de saúde na promoção de ambientes que valorizem a dimensão espiritual dos profissionais de enfermagem.

## Data Availability

Os dados da pesquisa estão disponíveis no corpo do artigo.
